# The Good Old Times

**Published:** 1881-09

**Authors:** 


### THE GOOD OLD TIMES.

I REMEMBER having seen a picture in *Punch* that represented several robust and aged Englishmen seated around a table, discussing their ale and the perversity of the times. They regretted the march of improvements and sighed for the days of "post coaches," wood fires and old-fashioned plum pudding. These vain but foolish regrets are not confined to superannuated Englishmen; such persons are to be found everywhere; they are the "camp followers" of that great and ever-increasing army of enterprising men that are compelling the universe to give up its secrets. These have done more for the advancement of the race in fifty years than has been accomplished in all the past ages; but with all their enterprise, with inventive genius that has astonished the world, no plan has yet been devised to silence the croakers. To the end of time they will doubtless kick against the forces that are impelling them forward, and vainly sigh for a return of the buried past. According to these, our grandfathers were a race of giants, whose forced toil and deprivations so toughened their constitutions that they lived as long as they pleased, and departed when they got ready, as a matter of choice, simply. The fact is that they were broken down by over-work and violations of sanitary laws, and went to their graves ten years earlier than the present generation. Then there is the rum-drinker. If he could only get a supply of the original stuff that preserved the glorious old fellows of the last century to green old age, what a blessing it would be to him and to the rising generation. This modern, adulterated stuff cuts and inflames, while that our grandfathers made was bland as milk and promoted longevity. Now, if whiskey is to be admitted among the "pleasures of memory," it is well to get at the truth about it. I at one time accepted the quite general belief that, although drinking was more common in the first half of the nineteenth century, deaths from drunkenness were comparatively few. I remember being greatly amused, when a boy, at the antics of a number of habitual drunkards who paraded the streets of my native village almost every afternoon; I remember their names, and how they looked and acted; they seemed to me like old men at that time, and as the years rolled on, and I would hear of the death of one and then another, until they were all gone, it never occurred to me that they could have died of anything but old age. I remember one man of whom I was always in mortal terror ; I kept him at a safe distance. This man was Asa S——, a wealthy farmer who lived near the village. He was a large man, and he used to ride into the village in a farm wagon, bareheaded, very drunk and very noisy. He seemed to be an old man ; and, when I heard one day that he was dead, it did not occur to me that whiskey had anything to do with his death. Years afterward I revisited my old home; curious to know what had become of those I had known in childhood, I sought information—in the best place to find it—the old burying ground. I came across the grave of old Asa S—— ; if the inscription on his tombstone told the truth, he died at the age of 26 ; and still it was that mild, pure, old-fashioned whiskey that killed him. I found the graves of nearly all these ancient topers, and not one had lived to be forty years old.—*Sic transit gloria.*

